# Degeneration of the mouse retina upon dysregulated activity of serum response factor

**Published:** 2011-04-29

**Authors:** Jenny Sandström, Peter Heiduschka, Susanne C. Beck, Ulrike Philippar, Matthias W. Seeliger, Ulrich Schraermeyer, Alfred Nordheim

**Affiliations:** 1 Department of Molecular Biology, Interfaculty Institute for Cell Biology, University of Tuebingen, Tuebingen, Germany; 2 Section of Experimental Vitreoretinal Surgery, University Eye Hospital of Tuebingen, Germany; 3 Division of Ocular Neurodegeneration, Centre for Ophthalmology, Institute for Ophthalmic Research, University of Tuebingen, Tuebingen, Germany

## Abstract

**Purpose:**

Our aim was to generate and phenotypically characterize a transgenic mouse line expressing a constitutively active variant of the transcription regulatory protein serum response factor (SRF), namely the SRF-VP16 protein. This new mouse strain has been registered under the designation *Gt(ROSA)26Sor^tm1(SRF-VP16)Antu^*. We found phenotypic changes upon ectopic expression of *SRF-VP16,* especially in the mouse retina.

**Methods:**

Using homologous recombination, we integrated an *SRF-VP16* conditional (i.e., “flox-STOP” repressed) expression transgene into the *Rosa26* locus of murine embryonic stem (ES) cells. These engineered ES cells were used to derive the *Gt(ROSA)26Sor^tm1(SRF-VP16)Antu^* mouse strain. Semiquantitative real-time PCR was used to determine expression of the *SRF-VP16* transgene at the mRNA level, both in young (P20 and P30) and adult (six months old) *Gt(ROSA)26Sor^tm1(SRF-VP16)Antu^* mice. We also investigated the transcript levels of endogenous *Srf* and several SRF target genes. Retinal function was tested by electroretinography in both young and adult mice. Morphological abnormalities could be visualized by hematoxylin and eosin staining of sectioned, paraffin-embedded eye tissue samples. Scanning-laser ophthalmoscopy was used to investigate retinal vascularization and degeneration in adult mice.

**Results:**

We show that the *SRF-VP16* mRNA is expressed to a low but significant degree in the retinas of young and adult animals of the *Gt(ROSA)26Sor^tm1(SRF-VP16)Antu^* mouse strain, even in the absence of Cre-mediated deletion of the “flox-STOP” cassette. In the retinas of these transgenic mice, endogenous *Srf* displays elevated transcript levels. Ectopic retinal expression of constitutively active SRF-VP16 is correlated with the malfunction of retinal neurons in both heterozygous and homozygous animals of both age groups (P20 and adult). Additionally, mislamination of retinal cell layers and cellular rosette formations are found in retinas of both heterozygous and homozygous animals of young age. In homozygous individuals, however, the cellular rosettes are more widespread over the fundus. At adult age, retinas both from animals that are heterozygous and homozygous for the *floxSTOP/SRF-VP16* transgene display severe degeneration, mainly of the photoreceptor cell layer. Wild-type age-matched littermates, however, do not show any degeneration. The severity of the observed effects correlates with dosage of the transgene.

**Conclusions:**

This is the first report suggesting an influence of the transcription factor SRF on the development and function of the murine retina. Ectopic *SRF-VP16* mRNA expression in the retinas of young animals is correlated with photoreceptor layer mislamination and impaired retinal function. At an advanced age of six months, degenerative processes are detected in *SRF-VP16* transgenic retinas accompanied by impaired retinal function. The *Gt(ROSA)26Sor^tm1(SRF-VP16)Antu^* mouse strain represents a genetic SRF gain-of-function mouse model that will complement the current SRF loss-of-function models. It promises to provide new insight into the hitherto poorly defined role of SRF in retinal development and function, including potential contributions to ophthalmologic disorders. Furthermore, using conditional Cre-mediated activation of SRF-VP16, the described mouse strain will enable assessment of the impact of dysregulated SRF activity on the physiologic functions of various other organs.

## Introduction

In the mouse eye, the retina is composed of various cell types that are strictly organized into distinct cellular layers, all descending from the primitive central nervous system. The structural organization of photoreceptor cells, interneurons, and ganglion cells into these layers is completed postnatally. In addition to cell differentiation, this lamination process includes neuronal migration, neurite outgrowth, axonal guidance, and synaptic targeting [1-3]. Previous reports have shown that the ubiquitously expressed transcription factor serum response factor (SRF) plays an important role in all of these processes [4-8].

SRF binds CC(AT)_6_GG DNA sequences (called CArG-boxes), found near promoter regions of SRF target genes [9,10]. The SRF target genes *c-fos*, *junB*, and *Egr-1* belong to the immediate early gene family and the *Srf* gene itself has CArG-boxes in its promoter region [11-14]. SRF is an important regulator of the actin cytoskeleton, driving transcription of the *Actb* and *Vcl* genes, among others [15-17]. The SRF-mediated effects on the actin cytoskeleton have been studied in both murine embryonic stem (ES) cells and in mice with different tissue-specific conditional *Srf* deletions [18].

Deletion of full length SRF in ES cells results in impairment of actin stress fiber formation and focal adhesion assembly [7]. The observed defects can be rescued by transfection of a construct expressing a constitutively active variant of SRF, SRF-VP16. This fusion protein has been reported to drive transcription of *Srf* target genes in vitro, and the compensatory effect of SRF-VP16 is stronger in cells depleted of endogenous *Srf* than in ES cells still expressing endogenous *Srf* [7,19]. Interestingly, neurons expressing endogenous wild-type SRF show an increase in the expression of SRF target genes upon additional ectopic expression of SRF-VP16 [20].

Conditional deletion of *Srf* in neurons of the murine forebrain revealed a crucial role of SRF in neuronal migration, neurite outgrowth, and axonal guidance. Impairment in the outgrowth activity of SRF-depleted neurons in vitro could be overcome by treatment with SRF-VP16 [6]. The same study also showed the requirement of properly functioning SRF for correct axonal targeting in the brain. Neuronal SRF depletion in vivo resulted in an aberrant circuit assembly within the hippocampus due to misguided mossy fibers [6].

Further investigations of the neuron-specific ablation of SRF showed the importance of SRF for cell autonomous neuronal migration. Neurons depleted of SRF in vivo fail to migrate along the rostral migratory stream to the olfactory bulb. Instead, SRF-depleted neurons accumulate in the subventricular zone [4].

To further complement our studies of SRF function in vivo, we generated a mouse line permitting the in vivo expression of the constitutively active SRF-VP16 protein. For this purpose, we engineered a transgenic mouse carrying a genomic integration of a *floxSTOP/SRF-VP16* sequence within the genomic ROSA26 locus. This transgene should allow for the Cre-mediated, conditional expression of the SRF-VP16 fusion protein under transcriptional control of the endogenous ROSA26 gene promoter. We here present an initial characterization of the *Gt(ROSA)26Sor^tm1(SRF-VP16)Antu^* mouse line. We demonstrate that this line displays an ectopic expression of SRF-VP16 in the eye, resulting in a pronounced retinal phenotype. This effect was observed in the absence of Cre-mediated recombination, due to transcriptional “leakiness” of the STOP cassette. Upon ectopic *SRF-VP16* retinal expression, P20 animals displayed retinal mislamination and cellular rosette formation in both heterozygous and homozygous retinas. Adult animals with ectopic retinal *SRF-VP16* expression showed degeneration of the retina; in particular, the photoreceptor cell layer was affected. Furthermore, these defects displayed dependence on dosage of the transgene, as seen at both ages and in severity regarding the disturbance of retinal function, retinal cellular rosette formation, and retinal degeneration.

This is the first study to suggest a correlation between dysregulated SRF activity and malformations of the postnatal retina. The mouse model presented here offers a complementary genetic approach to the existing SRF knockout systems, and therefore may enable new insight into the potential roles of SRF in retinal development and function.

## Methods

### Generation of the *Gt(ROSA)26Sor^tm1(SRF-VP16)Antu^* mouse strain

We aimed for conditional expression of a constitutively active variant of the SRF protein in mice, in a temporally and spatially controlled fashion. For this purpose, a DNA-targeting construct for genomic integration into the ubiquitously expressed *ROSA26* locus was generated; this encoded the *SRF-VP16* fusion protein under the control of a deletable STOP cassette. The human SRF coding sequence of the targeting construct was composed of a C-terminally truncated human *SRF* cDNA (encoding amino acids 1–412) which had the endogenous transactivation domain replaced by the transcription activation domain (C-terminal 80 amino acids) of the herpes simplex virus protein VP16, as previously described [21]. To allow for the conditional expression of the *SRF-VP16* fusion gene, a STOP cassette composed of four SV40 polyadenylation signals (SV40 polyA) and a neighboring Puromycin resistance gene [22] were introduced upstream of the *SRF-VP16* coding sequence. The Puromycin resistance gene was expressed under the control of the phosphoglycerate kinase (PGK) promoter and its transcriptional direction was opposite to that of *SRF-VP16*. In addition, a splice acceptor site was introduced 5′ of the STOP cassette and the *SRF-VP16* sequence, to achieve proper splicing and expression of the transgene after removal of the STOP cassette. The STOP cassette was flanked by two loxP sites, thereby enabling its conditional removal upon expression of active Cre recombinase. We refer to this conditional SRF-VP16 expression construct as *floxSTOP/SRF-VP16*.

**Figure 1:**
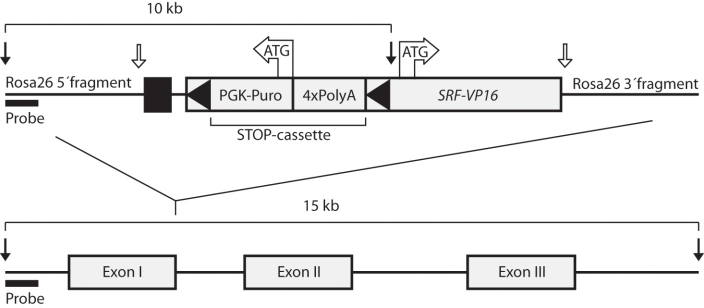
Schematic representation of the *Rosa26-floxSTOP/SRF-VP16* DNA targeting construct. The *Rosa26-floxSTOP/SRF-VP16* DNA targeting construct (upper) and its integration site in the wild-type genomic *Rosa26* locus (lower) are shown. Black arrows indicate EcoRI restriction sites, applied to Southern blot detection of the targeting construct (10 kb band) and the wild-type *ROSA26* locus (15 kb band), using the probe indicated. White arrows indicate XbaI restriction sites generated upon the insertion of the *floxSTOP/SRF-VP16* construct into the ROSA26–1 vector. The black box depicted upstream of the Puro-PGK cassette represents the splice acceptor. Black triangles indicate the two loxP sites flanking the STOP cassette.

The *floxSTOP/SRF-VP16* construct was cloned into the XbaI site of the pROSA26–1 vector [23], thereby generating the *Rosa26-floxSTOP/SRF-VP16* targeting construct (Figure 1, upper panel). This could be integrated by homologous recombination into the genomic *Rosa26* locus (Figure 1, lower panel). Murine R1 ES129 embryonic stem cells (SVP129 background) were electroporated with the linearized targeting vector. Following selection with puromycin, surviving ES cell clones were screened by genomic PCR for integration within the ROSA26 locus. Genomic DNA from positive clones was digested with EcoRI and further tested by Southern blotting for the presence of a 10 kb fragment corresponding to transgene integration within the targeted locus (not shown) [24]. Recombinant ES cell clones were injected into C57BL/6 blastocysts to generate chimeras that were bred further for germ line transmission, resulting in the *Gt(ROSA)26Sor^tm1(SRF-VP16)Antu^* mouse strain (registered under this name with the Mouse Genomic Nomenclature Committee, Jackson Laboratory, MGI accession number 4838411). Mice carrying one allele for *Rosa26-floxSTOP/SRF-VP16* were denoted heterozygous (het) and animals with two *Rosa26-floxSTOP/SRF-VP16* alleles were referred to as homozygous (hom). Wild-type mice without any rearrangement of the *Rosa26* locus were denoted *Rosa26* wild-type (wt). Genotypes were determined by genomic PCR on DNA preparations from tail biopsies using the following primers; *ROSA-*fw (5′-GGA GGC AGG AAG CAC TTG CTC TCC-3′) and *ROSA-*rev (5′-CAC CAG GTT AGC CTT TAA GCC TGC CC-3′) for the *Rosa26* wild-type locus and *ROSA-*fw (5′-GGA GGC AGG AAG CAC TTG CTC TCC-3′) and *PURO-*rev (5′-GAA CGA GAT CAG CAG CCT CTG TTC CAC-3′) for *Rosa26-floxSTOP/SRF-VP16*. Note that the endogenous *Srf* locus was unaffected by integration of the *Rosa26-floxSTOP/SRFVP16* transgene into the *Rosa26* locus.

Animal housing and handling was conducted in accordance with the Federation of European Laboratory Animal Science Associations and the local ethics committee (Regierungspräsidium Tübingen) approved all experiments presented in this study.

### Reverse-transcription and semiquantitative real-time PCR analysis

Total mRNA was prepared from freshly isolated tissues of mice aged P20 to P24, as well as from six-month-old animals, using the RNeasy Kit (Qiagen, Düsseldorf, Germany), and subsequently treated with DNaseI (Roche, Rotkreutz, Switzerland). cDNA was synthesized from 1 μg of total mRNA and reverse-transcription was performed (M-MLV Reverse Transcriptase, Promega, Madison, WA) following the manufacturer’s instructions. One-twentieth of the reverse transcription reaction was used for a 15 μl PCR reaction, and real-time amplification was monitored using Sybr® Green (Applied Biosystems, Foster City, CA) technology [7]. Glyceraldehyde 3-phosphate dehydrogenase (*Gapdh*) mRNA was used as internal control for each sample and all reactions were run in triplicate. Primers *GAPDH-*fw (5′-TGG ATC TGA CGT GCC GC-3′) and *GAPDH-*rev (5′-TGC CTG CTT CAC CAC CTT C-3′) were used for *GAPDH* message, *Srf-*fw (5′-TGT GCA GGC CAT TCA TGT G-3′) and *Srf-*rev (5′-ACA GAC GAC GTC ATG ATG GTG-3′) for the 3′ part of the endogenous *Srf* message, and *VP16-*fw (5′-CTT AGA CGG GCA AGT G-3′) and *VP16-*rev (5′-CCC AAC ATG TCC AGA TCG AAA-3′) for the *VP16* part of the *SRF-VP16* message. Statistical significance was calculated using the Student’s *t*-test and standard deviations are presented in standard error of the mean (SEM).

### Electroretinography

Electroretinography (ERG) measurements were performed with P30 mice as well as six-month-old mice (adult). Of the fifteen P30 mice, 5 were wild-type, 5 were *floxSTOP/SRF-VP16* (het), and 5 were *floxSTOP/SRF-VP16* (hom). Of the fourteen adult mice, 4 were wild-type, 5 were *floxSTOP/SRF-VP16* (het), and 5 were *floxSTOP/SRF-VP16* (hom).

Animals were dark-adapted over a period of at least 24 h. They were anesthetized by an intraperitoneal injection of a mixed ketamine/xylazine solution (120 mg/kg ketamine, 10 mg/kg xylazine). The cornea of the eye was desensitized with a drop of Novesine (Novartis Ophthalmics, Basel, Switzerland). The upper eyelids were retracted slightly by a surgical silk thread. To keep body temperature constant, animals were placed onto a heated platform (37 °C) during the measurements. Gold-wire-ring electrodes placed onto the corneas of both eyes served as working electrodes and a gold-wire-ring electrode placed in the mouth served as a reference electrode. A stainless steel needle electrode was inserted into the tail of the animals for grounding. The pupils were dilated with a drop of tropicamide (Novartis Ophthalmics). All manipulations were performed under dim red light, which was switched off after finishing all the stages of animal preparation. After an additional 5 min to allow the pupils to dilate, measurement was started using the commercial RetiPort32 device from Roland Consult Systems (Brandenburg, Germany).

Standard ERG measurements were performed, with scotopic flash ERG at up to eight different light intensities from 0.0003 to 100 cd⋅s/m^2^, an additional run for scotopic oscillatory potentials at 100 cd⋅s/m^2^, photopic 30 Hz flicker at 3 cd⋅s/m^2^ after 10 min of light adaptation, photopic flash ERG, and photopic oscillatory potentials. The light intensity used for the flashes in the photopic ERG measurements was 100 cd⋅s/m^2^.

The time of measurement was 160 ms, with 512 data points per measured waveform. The analog filters of the ERG device were set to the frequency ranges of 0.5 to 200 Hz for both scotopic and photopic flash ERG, 50 to 500 Hz for oscillatory potentials, and 10 to 50 Hz for 30 Hz flicker. In addition, the waveforms of the oscillatory potentials were digitally filtered offline using a digital signal processing (DSP) filter included in the software of the ERG device (−15 dB for f<10 Hz). The amplitudes of a-waves were measured from the baseline to the bottom of the a-wave trough, whereas b-wave amplitudes were measured from the bottom of the a-wave trough to the peak of the b-wave. ERG measurements were performed simultaneously on both eyes in each animal, and the eye giving the better parameters was chosen for data evaluation.

### Histological methods

Eyes from P20-to-P24 or six-month-old (P180) animals were fixed for 4 h in Davidson’s fixative (PBS; 137 mM NaCl, 2.7 mM KCl, 10mM Na_2_HPO_4_ and 2 mM KH_2_PO_4_, containing 6% formaldehyde, 32% ethanol, 11% acetic acid, and 5% sucrose) before further processing. Histological examination of the retina was done on 4 μm sections of paraffin-embedded eyes, mounted on Superfrost Plus slides (Langenbrinck, Emmendingen, Germany). Sections were stained with hematoxylin and eosin; residual stain was removed by washing under running tap water, followed by dehydration and mounting in Entellan® Neu (Merck KGaA, Darmstadt, Germany) under coverslips.

### Scanning-laser ophthalmoscopy

Scanning-laser ophthalmoscopy (SLO) was performed according to previously reported procedures [25]. Briefly, mice were anesthetized by subcutaneous injection of ketamine (66.7 mg/kg) and xylazine (11.7 mg/kg). After anesthesia, pupils were dilated with tropicamide eye drops (Mydriaticum Stulln, Pharma Stulln, Stulln, Germany). SLO imaging was performed with a Heidelberg Retina Angiograph (HRA I) equipped with an argon laser featuring two wavelengths (488 nm and 514 nm) in the short wavelength range and two infrared diode lasers (795 nm and 830 nm) in the long wavelength range.

The laser wavelength used for fundus visualization was 514 nm (red-free channel). The 488 nm wavelength was used for fundus autofluorescence (FAF) analysis. Additionally, the 488 nm and 795 nm lasers were used for fluorescein (FL) angiography (FLA) and indocyanine green (ICG) angiography (ICGA), respectively. FLA was performed using a subcutaneous injection of 75 mg/kg bodyweight FL-Na (University Pharmacy, University of Tübingen, Germany) and ICGA following a subcutaneous injection of 50 mg/kg bodyweight ICG (ICG-Pulsion, Pulsion Medical Systems AG, Munich, Germany).

## Results

### Generation of transgenic mice allowing conditional expression of the constitutively active SRF-VP16 fusion protein

To establish a genetic model for the study of phenotypic effects elicited by conditional in vivo expression of a constitutively active variant (termed SRF-VP16) of the transcription factor SRF, we generated a transgenic mouse carrying an inducible *SRF-VP16* transgene in its genome (for details, see Materials and Methods). The obtained mouse line was named *Gt(ROSA)26Sor^tm1(SRF-VP16)Antu^*. The *SRF-VP16* transgene was composed of the coding sequence of the human *SRF,* where part of the SRF C-terminal transactivation domain was replaced by the herpes simplex virus VP16 transcriptional activation domain, as previously described by Dalton and Treisman (1992) [21]. Upstream of the *SRF-VP16* fusion cDNA, a STOP cassette was positioned; this was flanked by two loxP sites (Figure 1), permitting transcriptional read-through upon conditional Cre-mediated STOP cassette deletion. This deletion was achieved by crossing the *Gt(ROSA)26Sor^tm1(SRF-VP16)Antu^* strain with the CamKIIα-iCre mouse line [26] expressing Cre recombinase (data not shown). In this report, however, we focus on the effects observed upon *Rosa26-floxSTOP/SRF-VP16* transgene integration independent of Cre expression, since as detailed below, the presence of the genomic STOP cassette did not fully prevent expression of SRF-VP16. *Gt(ROSA)26Sor^tm1(SRF-VP16)Antu^* mice carrying the Rosa26-floxSTOP/SRF-VP16 transgene were born with the expected Mendelian distribution and their behavior (gait, feeding, and motor reflexes) was indistinguishable from that of their wild-type littermates. We did, however, observe a weight reduction in both hetero- and homozygous *Rosa26-floxSTOP/SRFVP16* mice of about 22% (p=0.0546, Student *t*-test) as compared to *Rosa26* wild-type littermate controls (data not shown). Despite the weight reduction, genomic integration of the *Rosa26-floxSTOP/SRF-VP16* allele did not affect either lifespan or fertility of the *Gt(ROSA)26Sor^tm1(SRF-VP16)Antu^* mice.

### Ectopic retinal expression of the *Rosa26-floxSTOP/SRF-VP16* transgene

**Figure 2:**
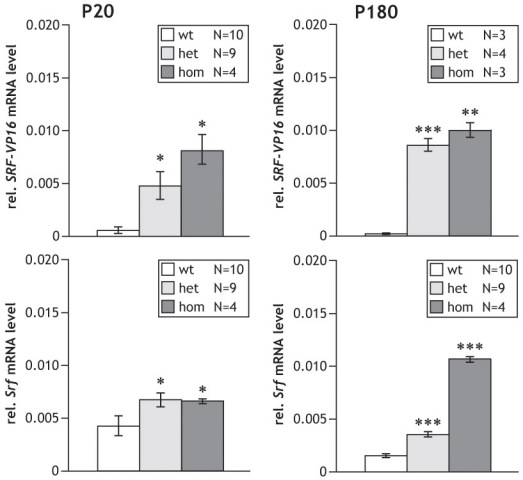
Retinal mRNA levels of *SRF-VP16* and endogenous *Srf* at ages P20 and P180. Real-time PCR quantification of relative *SRF-VP16* and endogenous *Srf* mRNA levels in the retinas of P20 and P180 mice. *SRF-VP16* RNA levels were only observed in retinas of *Rosa26-floxSTOP/SRF-VP16* transgenic mice, both heterozygous (het) and homozygous (hom) for the *SRF-VP16* transgene (upper panels). Expression of endogenous *Srf* was always elevated compared to wild-type mice in the presence of the transgene; this was especially pronounced in the transgenic P180 (hom) mice (lower panels). Error bars indicate SEM, *p<0.05, **p<0.01, and ***p<0.001, denoting significance as compared to wild-type mice. All transcriptional levels were normalized to *Gapdh*.

A relevant feature of transcriptional STOP cassettes is their “transcriptional tightness,” i.e., their ability to efficiently prevent the expression of the associated transgene. At the same time, removal of the STOP cassette should enable efficient expression of the transgene. We tested for potential “leakiness” of the STOP cassette and investigated mRNA expression of the *SRF-VP16* transgene in *Gt(ROSA)26Sor^tm1(SRF-VP16)Antu^* mice using semiquantitative real-time PCR. Expression was investigated in samples from the heart, liver, skeletal muscle, brain (cortex, hippocampus, and cerebellum), lens, and retina. Interestingly, *SRF-VP16* expression was found in the retina (Figure 2), while all of the other tissues investigated were negative for *SRF-VP16* mRNA expression (data not shown). *SRF-VP16* transcripts could already be detected in both hetero- and homozygous retinas at day P8 at very low levels (not shown); statistical evaluation was first possible at day P20 (Figure 2, upper left).

At the protein level, we were unable to detect SRF-VP16 protein expression by western blot analysis using anti-VP16 antisera on tissue lysates from P20-to-P24 retinas (data not shown). This is likely due to low SRF-VP16 expression levels and/or insufficient sensitivity of the antisera available. We also attempted to detect the *SRF-VP16* protein by immunohistochemical staining on P20 specimens using anti-VP16 antisera. However, no signal of SRF-VP16 immunoreactivity could be detected (data not shown). Endogenous SRF protein was ubiquitously expressed in all cells of the retina, and the observed ectopic expression of *SRF-VP16* mRNA did not manifest itself in an increased immunoreactivity of endogenous SRF (data not shown).

To further characterize the expression of the *Rosa26-floxSTOP/SRF-VP16* transgene in the retina, we analyzed transcript levels in adult animals at the age of six months (P180). At this age, comparable to P20, significant levels of *SRF-VP16* mRNA were observed in both hetero- and homozygous retinas (Figure 2, upper right). With P180 animals, we also tried to address retinal SRF-VP16 protein expression. However, as seen with the P20 retinas, western blotting of P180 retinal extracts also failed to reveal SRF-VP16 protein expression (data not shown). Similarly, the SRF-VP16 protein could not be detected by immunohistochemistry (data not shown).

Since SRF is known to autoregulate transcription of its own gene [14], we also investigated whether the *SRF-VP16* transgene influenced the expression levels of endogenous *Srf* mRNA. Indeed, we could observe a significant, 1.7 fold elevation of *Srf* mRNA expression in transgenic heterozygous and homozygous P20 retinas, as compared to wild-type retina levels (Figure 2, lower left). In adult tissue, we observed elevations in *Srf* mRNA levels that were 2.3 fold in heterozygous and 6.7 fold in homozygous retinas, as compared to wild-type levels (Figure 2, lower right). We also investigated the mRNA expression levels of other known SRF target genes, namely *Bcl2*, *Actb*, and *SMA*, but no significant change in expression levels in either of these genes could be observed (data not shown). We conclude that, in the retinal tissue of *Gt(ROSA)26Sor^tm1(SRF-VP16)Antu^* mice, the *Rosa26- floxSTOP/SRF-VP16* transgene is transcribed to low yet significant levels, giving rise to *SRF-VP16* mRNA. We interpret this RNA product to be due to “leaky” read-through transcription, since the presence of the nonrearranged STOP cassette was confirmed both by genomic PCR and semiquantitative real-time PCR on genomic DNA (not shown). Furthermore, the presence of *SRF-VP16* message correlated with elevated levels of endogenous *Srf* mRNA.

### Retinal malfunction upon ectopic SRF-VP16 activation

**Figure 3:**
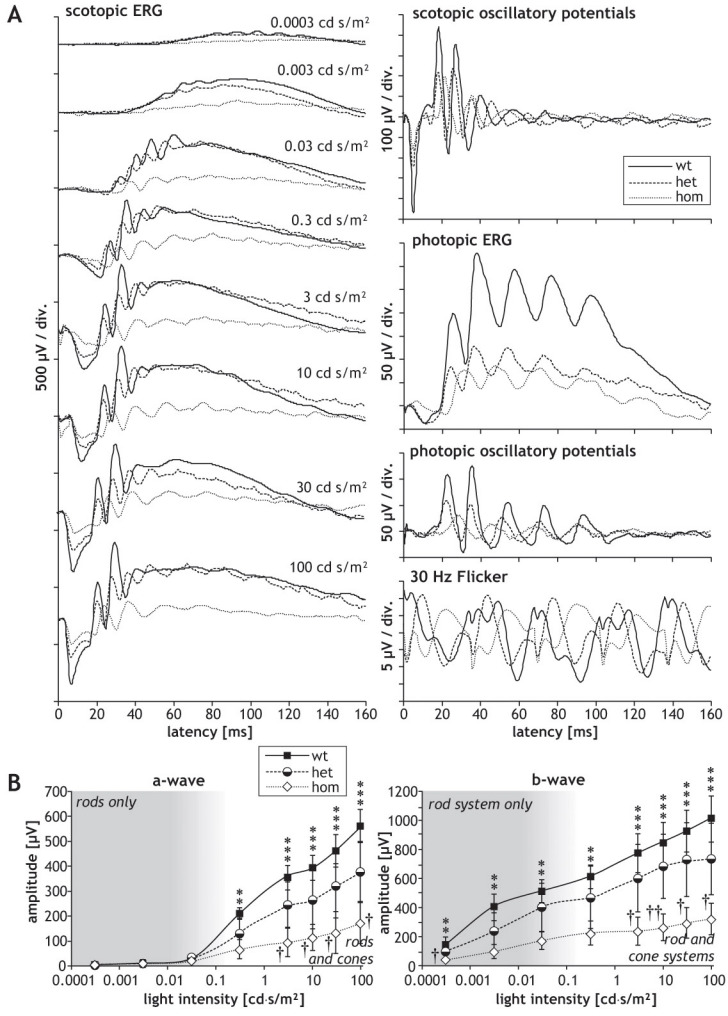
Graphs presenting electroretinographic measurements of animals at age P30. Electoretinographic (ERG) waveforms obtained in P30 (one-month-old) mice of the *Rosa26* (wt), *Rosa26-floxSTOP/SRF-VP16* (het), and *Rosa26-floxSTOP/SRF-VP16* (hom) genotypes. **A**: Typical waveforms are shown of scotopic and photopic ERGs, scotopic and oscillatory potentials, as well as the photopic 30 Hz flicker, as indicated. Note the different scaling. **B**: Values of amplitudes of scotopic a-waves and b-waves, depending on the intensity of light stimuli. Significances of differences between wild-type and homozygous mice are indicated by asterisks, and significances of the differences between heterozygous and homozygous mice is indicated by crosses (* or †p<0.05, ** or ††p<0.01, ***p<0.001).

**Figure 4:**
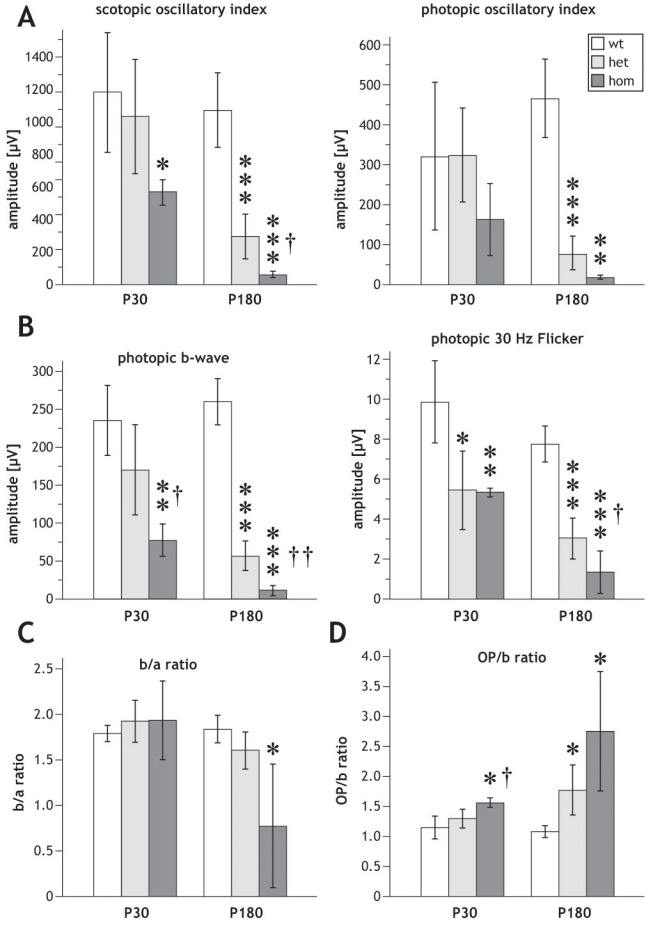
Quantification of electroretinographic parameters. Comparison of several electroretinographic (ERG) parameters obtained in P30 and P180 *Rosa26* wild-type (wt), *Rosa26-floxSTOP/SRF-VP16* (het), and *Rosa26-floxSTOP/SRF-VP16* (hom) mice, respectively, as indicated. **A**: Scotopic- (left) and photopic (right) oscillatory index. **B**: Photopic b-wave (left) and photopic 30Hz flicker (right). **C**: b/a ratio. **D**: OP/b ratio. Significances of differences between wild-type and transgenic mice are indicated by asterisks, and significances of the differences between heterozygous and homozygous mice are indicated by crosses (* or †p<0.05, ** or ††p<0.01, ***p<0.001).

Since *SRF-VP16* was shown to affect the neurite outgrowth of hippocampal neurons in vitro [6], we investigated potential functional consequences of retinal *SRF-VP16* mRNA expression in *Gt(ROSA)26Sor^tm1(SRF-VP16)Antu^* mice. For this purpose, we characterized retinal function by ERG measurements. Typical ERG waveforms obtained in wild-type P30 mice are shown in Figure 3A. The amplitudes of the scotopic ERG a-waves and b-waves from P30 heterozygous and homozygous mice are smaller than those of their wild-type littermates. The corresponding values of a-wave and b-wave amplitudes are shown in Figure 3B. The difference in amplitude between heterozygous and homozygous mice was not significant, whereas amplitudes obtained in homozygous mice were significantly smaller at higher light intensities than those obtained in wild-type mice. Amplitudes of scotopic and photopic oscillatory potentials of P30 heterozygous mice were similar to those of wild-type mice, whereas those of homozygous animals were reduced (Figure 3A; quantification in Figure 4A). The heterozygous P30 animals showed smaller photopic b-wave and photopic 30 Hz flicker amplitudes as compared to wild-type mice, and this reduction was even larger in homozygous animals (Figure 3A; quantification in Figure 4B). The ratio between the amplitudes of b-waves and a-waves, the so-called b/a ratio, was not different in the three strains (Figure 4C); in contrast, the ratio between the amplitudes of oscillatory potentials and b-waves, the OP/b ratio, was higher in homozygous animals than in wild-type animals (Figure 4D).

**Figure 5:**
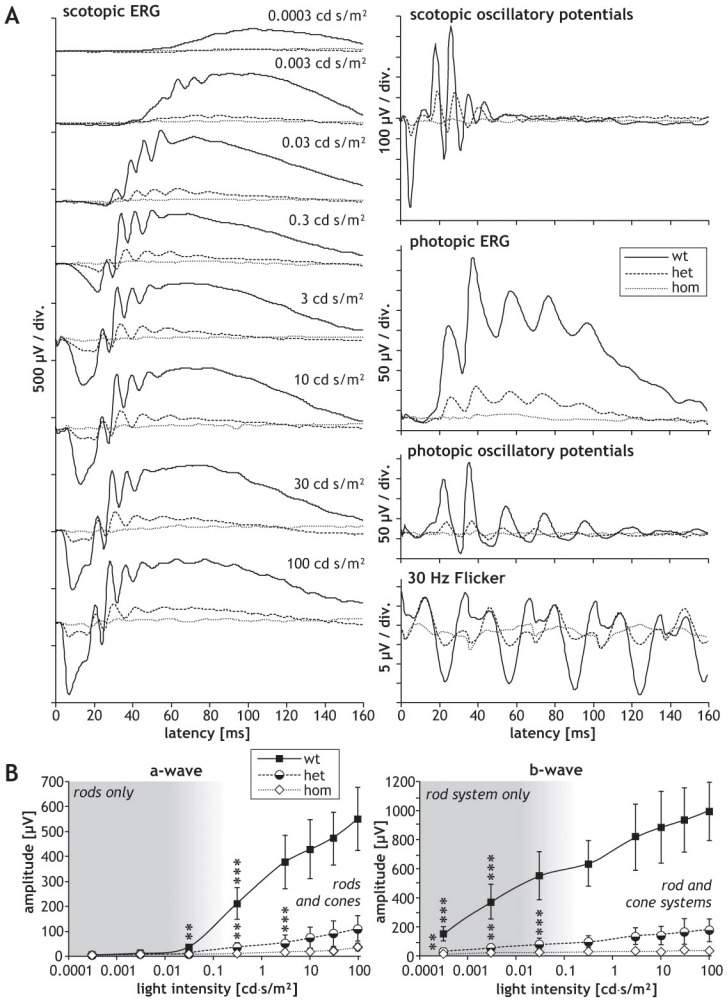
Graphs presenting electroretinographic measurements of animals at age P180. Electroretinographic (ERG) measurements obtained with P180 (six-month-old) mice of the *Rosa26* (wt), *Rosa26-floxSTOP/SRF-VP16* (het), and *Rosa26-floxSTOP/SRF-VP16* (hom) genotypes. **A**: Typical waveforms are shown of scotopic and photopic ERGs, scotopic and oscillatory potentials, as well as the photopic 30 Hz flicker, as indicated. Note the different scaling. **B**: Values of amplitudes of scotopic a-waves and b-waves depending on the intensity of light stimuli are shown. Amplitudes measured in wild-type mice are larger than amplitudes obtained in the mutants, with high significance over almost all light intensities (***p<0.001).

In P180 mice, we observed clear differences in the ERG waveforms and parameters of heterozygous and homozygous animals as compared to wild-type control animals. Furthermore, as compared to P30 animals, P180 wild-type animals also displayed waveforms typical of normal mice, as shown in Figure 5A. The ERG waveforms of the heterozygous animals were mildly affected, while those of homozygous animals were drastically affected. The scotopic a-wave and b-wave amplitudes in the heterozygous and homozygous mice were significantly smaller than in the wild-type animals. Moreover, the amplitudes of the homozygous animals were also significantly smaller than those of the heterozygous mice (Figure 5B). In addition, at this stage (P180), homozygous animals showed significantly diminished amplitudes of both scotopic and photopic oscillatory potentials as compared to heterozygous and wild-type animals, and the amplitudes of the heterozygous animals were also significantly reduced in comparison to wild-type controls (Figure 4A and Figure 5A). The reduction of photopic b-wave and photopic 30 Hz flicker amplitudes measured in heterozygous and homozygous mice compared to wild-type animals was even larger at P180 than the corresponding reduction at P30, and again significant (quantification in Figure 4B). No significant difference could be found in the b/a ratio between wild-type and heterozygous mice at P180 (Figure 4C), whereas it was markedly decreased in homozygous animals compared to wild-type mice (Figure 4C). The OP/b ratio was significantly increased in P180 heterozygous and homozygous animals compared to wild-type mice (Figure 4D).

In contrast to all the differences in amplitudes shown here, no significant differences in the latencies of a-waves, b-waves, or oscillatory potentials could be found, although the latencies obtained in homozygous mice had a slight tendency to be prolonged at both investigated ages (not shown).

### Photoreceptor mislamination in SRF-VP16 positive retinas

**Figure 6:**
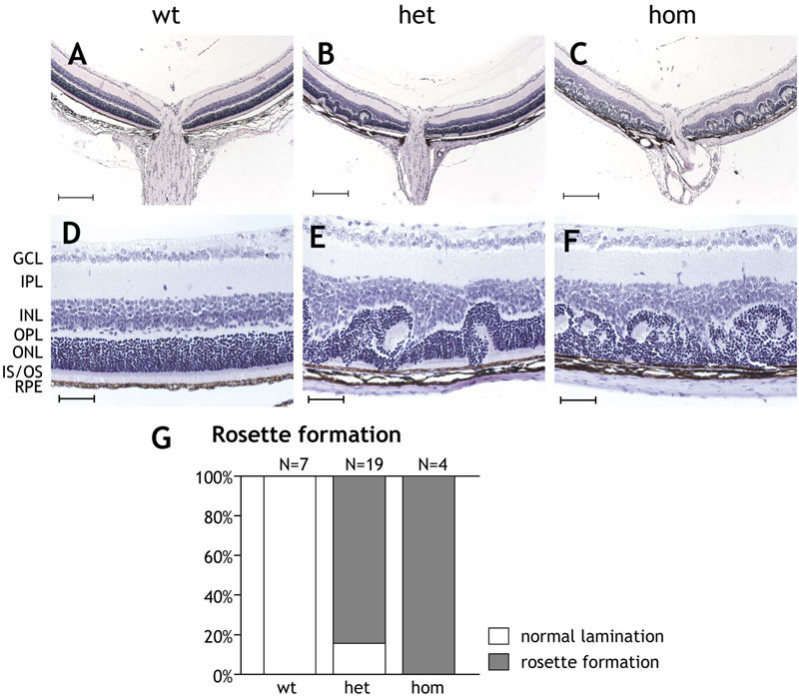
Rosette formations in the photoreceptor layer of P20 animals heterozygous or homozygous for the *Rosa26-floxSTOP/SRF-VP16* transgene. Histological sections are provided displaying rosette formations in the photoreceptor layers of *Rosa26- floxSTOP/SRF-VP16* heterozygous or homozygous P20 animals. **A**-**C**: Hematoxylin and eosin–stained sections are shown of retinas obtained from wild-type, heterozygous, and homozygous P20 animals. Scale bar represents 200 μm. **A**: Normal wild-type retina is displayed and compared to those of heterozygous (**B**) and homozygous (**C**) transgenic animals, showing the localization of rosette formation around the vicinity of the optic nerve. **D**-**F**: Magnifications are shown of regions of the retinas displaying normal (**D**) or dyslaminated (**E**, **F**) retinal layers. Normal mislamination in wild-type retinas (**D**) with the ganglion cell layer (GCL), inner plexiform layer (IPL), inner nuclear layer (INL), outer nuclear layer (ONL), inner and outer segments (IS/OS), and retinal pigment epithelium (RPE). Rosette formations to a varying extents are shown in heterozygous (**E**) and homozygous (**F**) P20 retinas. Scale bars represent 50 μm. **G**: Quantification of rosette formation is shown. Retinas displaying rosette formations were categorized as positive for rosette formation. Eighty-seven percent of the heterozygous animals showed rosette formation, while 100% homozygous animals displayed rosette structures.

We next investigated the morphological basis for the severe disturbances of ERG characteristics in hetero- and homozygous transgenic animals. Interestingly, upon histological examination, we observed a disordered lamination in the direct circumference of the optic nerve in both heterozygous and homozygous P20 retinas (Figure 6B,C, arrowheads). However, in the periphery, retinal lamination appeared normal in the eyes of both heterozygous and homozygous animals (Figure 6A-C). Larger microscopic magnifications of the disorganized areas revealed rosette formation in the photoreceptor cell layer (Figures 6E,F), where the inner and outer segments appeared encircled by photoreceptor cell nuclei constituting the outer nuclear layer segments. In some cases, formation of rosettes in heterozygous animals could be observed to the same extent as in homozygous animals (not shown), but in general, the majority of the heterozygous animals had less widespread rosettes compared to homozygous littermates. Statistical evaluation of all histological sections showed only a few heterozygous individuals with normal retinal lamination, comparable to those of wild-type retinas (Figure 6J). More than 80% of the heterozygous animals showed a significant degree of disorganization of the retinal layers (Figure 6H,J), while all homozygous animals examined displayed extensive formation of rosettes (Figure 6I,J). To exclude disturbances due to changes in the number of photoreceptor cell nuclei, the numbers of photoreceptor nuclei rows of the outer nuclear layer were counted. We did not observe any difference in between the wild-type animals and the hetero- or homozygous transgenic animals (data not shown).

### Retinal degeneration in adult SRF-VP16 expressing mice

**Figure 7:**
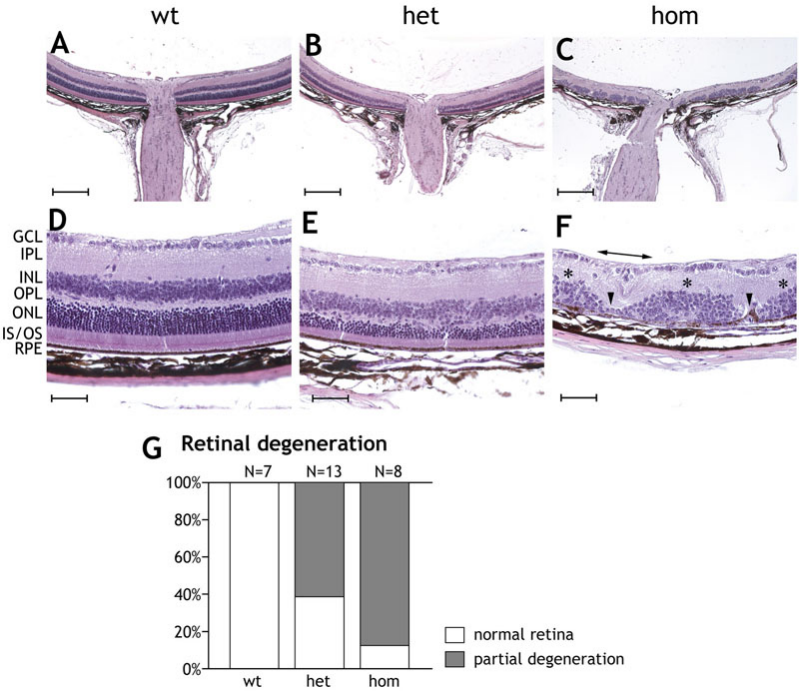
Retinal degeneration in P180 mice heterozygous or homozygous for the Rosa26-floxSTOP/SRF-VP16 transgene. Histological sections of P180 retinas are shown displaying degenerative processes in *Rosa26-floxSTOP/SRF-VP16* heterozygous (het) and homozygous (hom) animals. **A**-**C**: Hematoxylin and eosin staining. Note the scattered degeneration in the posterior part of heterozygous and homozygous retinas. Peripheral regions show no degeneration. Scale bars represent 200 μM. **D**-**F**: Higher magnifications of P180 retinas are displayed. Wild-type retina (**D**) showing ganglion cell layer, inner plexiform layer, inner nuclear layer, outer nuclear layer, inner and outer segments and retinal pigment epithelium (RPE). Degeneration of the photoreceptor layer in adult heterozygous (**E**) and homozygous (**F**) retinas is shown. In (**F**), arrowheads point to regions of complete degeneration of the inner and outer nuclear layers, whereas inner nuclear layers can be found at least partially in other places (asterisks). Occasionally, even the ganglion cell layer can no longer be found (indicated by horizontal double-headed arrow). Scale bars represent 50 μM. **G**: Quantification of individual mice displaying retinal degeneration in histological sections. Retinas displaying areas in the outer nuclear layer with eight or fewer layers of photoreceptor nuclei were classified as having “partial retinal degeneration.” Sixty percent of the heterozygous animals and over 80% of the homozygous animals showed partial degeneration.

Disorganization of retinal morphology was also found in the eyes of six-month-old (P180) heterozygous and homozygous *Gt(ROSA)26Sor^tm1(SRF-VP16)Antu^* mice (Figure 7). The most striking finding at this stage was a clear retinal degeneration in the vicinity of the optic nerves of heterozygous and homozygous transgenic animals (Figures 7B,C), compared to retinas of wild-type animals, which showed normal photoreceptor layers (Figure 7A). Little or no degeneration was found in more peripheral parts of the retina. The degeneration observed in P180 animals appeared to originate from areas equivalent to those that displayed rosette formation in P20 mice (Figure 6). Magnification of the degenerating areas revealed a partial degradation (eight rows or less) up to complete absence of the photoreceptor layers in heterozygous retinas (Figure 7E). In homozygous retinas, sites of degeneration were also scattered over the posterior part of the eye. Here, only parts of the inner nuclear layer, the inner plexiform layer, and the retinal ganglion cell layer remained detectable, as shown in Figure 7F. Moreover, the retinal pigment epithelium (RPE) was also disturbed at several sites. Statistical assessment of histological sections revealed that approximately 60% of the heterozygous animals showed degeneration in some regions of the retina, while more than 80% of the homozygous animals displayed degeneration (Figure 7G).

### SLO on six-month-old *Gt(ROSA)26Sor^tm1(SRF-VP16)Antu^* mice

**Figure 8:**
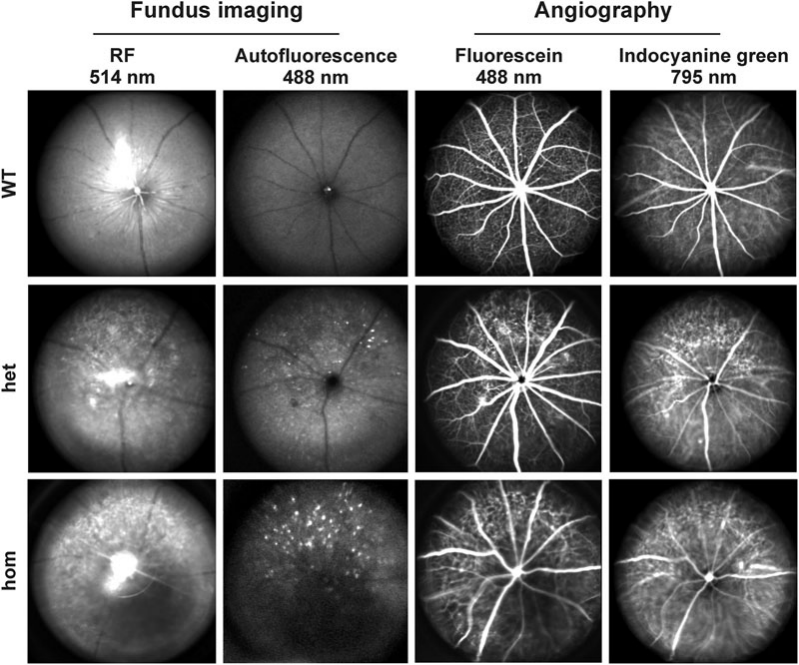
Scanning-laser ophthalmoscopy (SLO) imaging. Eyes from *Rosa26* (wt; upper row), *Rosa26-floxSTOP/SRF-VP16* (het) (middle row), and *Rosa26-floxSTOP/SRF-VP16* (hom; bottom row) animals at six months of age (P180) were investigated. Fundus imaging with RF at 514 nm excitation (left column) and autofluorescence at 488 nm laser wavelengths (middle left column) display dramatic changes in fundus appearance, structure of the nerve fiber layer and accumulation of autofluorescent lipids in genetically modified animals compared to wild-type animals. Retinal degenerative processes can also be inferred from enhanced angiographic deep layer (choroidal) signals using fluorescein (middle right column) and indocyanine green (right column) angiography, as clearly detected in both heterozygous and homozygous animals.

To further elucidate phenotypic changes in the retinas of six-month-old *Gt(ROSA)26Sor^tm1(SRF-VP16)Antu^* mice and to investigate whether vascular defects could be involved in the observed retinal degeneration, SLO [25] was performed (Figure 8). This analysis included native, red-free funduscopy at a wavelength of 514 nm to display retinal vessels and the nerve fiber layer (Figure 8, left column). Second, FAF analysis at 488 nm was applied (Figure 8, middle left column). FAF revealed retinal vessel structures as shadows embedded within a general diffuse “glow,” permitting the simultaneous visualization of pathologically enhanced autofluorescence [25]. Third, retinal and choroidal vessels were analyzed by FLA. Whereas the large retinal vessels and capillaries can be displayed in detail by FLA using fluorescein (FL) (Figure 8, middle right column), both retinal and choroidal vessels can be imaged when ICGA is applied (Figure 8, right column). In comparison to wild-type mice (Figure 8, upper row), SLO analysis of heterozygous (middle row) and homozygous animal eyes (lower row) revealed various changes in fundus appearance, which might be directly correlated with retinal degenerative processes. In 514 nm funduscopic images, large patchy areas were clearly visible in transgenic eyes (Figure 8, left column). FAF analysis revealed the pathological accumulation of autofluorescent material (likely derived from lipofuscin-containing lipids) in these areas (Figure 8, middle left column). In both heterozygous and homozygous animals, angiography with either FL (Figure 8, middle right column) or ICG (Figure 8, right column) revealed enhanced pathological visibility into deeper layers of the retina. Such sites of visibility of deeper retinal layers are not detectable in *Rosa26* wild-type animals. Moreover, large retinal blood vessels, as well as small capillaries, appeared to be more disturbed in mutant mice, particularly homozygous animals.

## Discussion

### Generation of SRF-VP16 expressing mice

In this report, we describe the first generation of a mouse line permitting conditional expression of a constitutively active variant of the transcription factor SRF. The line *Gt(ROSA)26Sor^tm1(SRF-VP16)Antu^* carries the *SRF-VP16* transgene within the ubiquitously expressed genomic *Rosa26* locus (Figure 1). Since transgene expression is impaired by the presence of a “floxed” STOP cassette, the desired conditional expression of the transgene can be activated by Cre-mediated deletion of the STOP cassette. We have shown that the induction of *SRF-VP16* expression occurred when the above transgenic line was bred with mice expressing the Cre recombinase cell type-specifically in neurons of the forebrain and hepatocytes of the liver (manuscripts in preparation). We therefore expect the mouse line described here to provide a useful tool for a variety of different genetic studies on SRF function.

The current report, however, focuses on the unexpected finding that the *Rosa26-floxSTOP/SRF-VP16* transgene is expressed selectively, at low constitutive levels, in retinal tissue. This expression is observed in the absence of Cre-mediated deletion of the STOP cassette. We can show in both one- and six-month-old animals that “leaky” expression of *SRF-VP16* mRNA occurs selectively in the retina, but not in the other tissues tested, including the heart, skeletal muscle, brain (cortex, hippocampus, and cerebellum), and lens. The STOP cassette used here has previously been reported to be tight [22], although the general phenomenon of “leakiness” of STOP cassettes has been discussed extensively [27-29]. Given the low basal levels of the transgenic transcript, and consequently our inability to visualize the SRF-VP16 protein, we presently do not know which cells of the retina express the *SRF-VP16* transgene, nor can we currently pinpoint the exact time of onset of its expression.

### Phenotypic consequences of retinal SRF-VP16 expression

Here, we describe the phenotypic changes displayed in the eyes of the *Gt(ROSA)26Sor^tm1(SRF-VP16)Antu^* mice, as observed upon leaky expression of the *SRF-VP16* transgene in the absence of Cre-mediated recombination. In P20 retinas of hetero- and homozygous transgenic animals, we observed not only significant transcript levels of *SRF-VP16*, but also increased levels of endogenous *Srf* mRNA (Figure 2). Since *SRF-VP16* has previously been reported to drive transcription of SRF target genes [7,20,30-32], including *Srf* itself [14], the increase in *Srf* mRNA is consistent with our expectations. This elevated expression of endogenous *Srf* in hetero- and homozygous retinas is maintained at an advanced age (six months), in a pattern suggesting the gene dosage dependence of the *SRF-VP16* allele (Figure 2).

Ectopic retinal expression of constitutively active SRF-VP16 is correlated with severe morphological abnormalities of the retina, including retinal mislamination and cellular rosette formation of both heterozygous and homozygous P20 transgenic animals. SRF has previously been shown to play a role in proper neuronal assembly in the postnatal hippocampus. The mossy fibers of SRF-depleted hippocampi fail to segregate into their proper layers and show fiber growth inside the pyramidal layer [6]. The disorganization of the photoreceptor layer in the P20 hetero- and homozygous animals, shown in Figure 6, indicates that the *SRF-VP16* transcript has an effect on the lamination of the postnatal retina. This suggests that regulated SRF activity is not only important for correct axonal guidance, but might also play a role in neuronal lamination, as recently demonstrated [33].

Furthermore, SRF has been shown to play a vital role in neuronal cellular motility, most likely due to its impact on actin cytoskeletal dynamics [4,8]. Neurons deficient in SRF show reduced capacity to migrate in vivo and axonal guidance is disturbed in vitro. Furthermore, SRF-ablated neurons treated with SRF-VP16 show abnormal axonal sprouting, displaying significantly longer protrusions compared to those of wild-type neurons [6]. These previously reported effects of SRF on neuronal motility and migration also suggest a possible role for SRF regarding the correct assembly of the neurons of the mouse retina. Whether the rosette formations we observed in the *SRF-VP16* positive retinas were due to “overmigration” or to incorrect neuronal guidance is still unclear, and this awaits further investigations into potential changes in cell proliferation and survival.

Another vital role of SRF regards its involvement in focal adhesion assembly, since murine embryonic stem cells ablated of SRF fail to adhere properly [7]. The same report also suggested the importance of SRF for directed migration, as exerted by its contribution to focal adhesion assembly. A speculative explanation is that *SRF-VP16*-positive retinal neurons hold incorrect contacts to extracellular structures or neighboring cells, and therefore are unable to position themselves in appropriate layers. However, since the exact spatiotemporal expression pattern of the SRF-VP16 read-through message has yet to be determined, we cannot currently distinguish between the observed rosette formation due to SRF-VP16 expression in neurons or in other cells of the retina.

### Disturbances of retinal function upon SRF-VP16 expression

ERG is a sensitive tool to detect impairments of retinal function. Such impairments can be observed in many pathological conditions before morphological or histological signs of the disease become visible. *Srf* has previously been shown to be important for murine neuronal function and maintenance in vivo [4,5,34,35]. Therefore, we first performed ERG in one- and six-month-old animals to check whether there were any effects of increased transcript levels of *SRF-VP16* on neuronal function in the retinas of young and adult mice.

In wild-type mice, normal electroretinograms could be recorded at both studied ages. In contrast, ERG amplitudes were reduced in all mutant mice. The degree of reduction varied in different animals, in clear correlation with the degree of structural abnormalities. This was especially the case in the heterozygous animals, as revealed by histological analysis. In one of the one-month old heterozygous mice, ERG amplitudes reached normal values, whereas ERG amplitudes were drastically reduced in all other heterozygous mice.

No significant changes were found in the latencies of scotopic and photopic a- and b-wave, and also not in the latencies of the oscillatory potentials. Possibly, the disturbances in the retinal structure lead only to a quantitative decrease of the retinal function, as the mechanisms of phototransduction and transmission of nerve signals appear to be unaffected. The similarities in the b/a ratios between wild-type mice and heterozygous mice indicate that the decrease of b-wave amplitudes has its origin in a decreased input from the photoreceptors. Interestingly, even the amplitudes of oscillatory potentials were not greatly affected in heterozygous mice, indicating an intact neuronal circuitry in the inner retina. This is in line with the histological findings in the heterozygous mice, which show occasional rosettes in the photoreceptor layer and virtually intact inner neuronal layers.

At the age of six months, the ERG amplitudes measured in both heterozygous and homozygous mice were drastically reduced. As seen in the corresponding waveforms, retinal activity was almost undetectable in homozygous mice at this point (Figure 5A). Again, latencies were not affected significantly. In contrast to the situation in one-month-old mice, amplitudes of oscillatory potentials were clearly decreased in the mutant mice, as were all other amplitudes. Therefore, the degenerative processes appear to have also reached the inner retina at the age of six months. Indeed, histological inspection shows strong disturbances not only in the photoreceptor layer, but also in the inner neuronal layers. Although the decreased amplitudes of oscillatory potentials show that degeneration has an effect on the inner neurons and their circuitry, Müller cells and bipolar cells seem to be affected even more, as can be deduced from the clearly decreased b/a ratios and the significantly increased OP/b ratios in the mutant animals.

### Correlation of retinal dysfunction and cellular rosette formation

There are some links made in the literature as to how rosette formation might be connected to disturbed ERG signals. One report shows that retinitis pigmentosa patients with photoreceptor rosette formations have nearly extinct ERG amplitudes [36], similar to what we observe in the adult six-month-old heterozygous *Gt(ROSA)26Sor^tm1(SRF-VP16)Antu^* mice. Mears et al. [37] described another mutant mouse line showing drastically reduced ERG amplitudes, where the neuronal retina leucine zipper protein has been deleted. Here, homozygous animals also display widespread rosette formations accompanying the disturbed ERGs. In the eyes of homozygous mice expressing *SRF-VP16*, ERG signals seems to be drastically decreased in adult animals compared to one-month-old mice. This observation has also been reported in several other mutant mouse strains displaying photoreceptor rosettes’ age-dependent decreases in ERG amplitudes [38]. In the latter study, it was proposed that the formation of the rosettes could be linked to the integrity of the outer limiting membrane or the interaction between photoreceptors and Müller cell microvilli. In the *Gt(ROSA)26Sor^tm1(SRF-VP16)Antu^* mice, one could speculate that rosette formation contributes to the disturbed function of the photoreceptor signaling and that this malfunction in turn leads to the loss of photoreceptors observed in adult mice.

### Retinal degeneration in adult mice upon expression of SRF-VP16

Adult retinas of six-month-old (P180) hetero- and homozygous transgenic SRF-VP16 animals displayed severe degeneration. In particular, the photoreceptor cell layer was reduced to half thickness in heterozygous and to complete absence in homozygous transgenic mice (Figure 7). The severity of the observed effects therefore correlated with transgene dosage. Furthermore, in both types of P180 transgenic animals, a severe destruction of the RPE was apparent. These effects appeared to represent a stage of advanced progression with regard to the defects seen in P30 mice (Figure 6). SLO was applied to characterize the degenerated eyes of P180 transgenic mice within the living animal. Fundus imaging (red-free and autofluorescence) and angiography (FLA and ICGA) independently gave clear indication of severe retinal degeneration. Degeneration of photoreceptors is often accompanied by an accumulation of autofluorescent, retinoid-containing debris material in the subretinal space [39]. The retinoids are derived from photoreceptor outer segment breakdown products that contain the visual pigment chromophore 11-cis-retinal. Autofluorescent debris signals were visibly scattered in the eyes of heterozygous transgenic mice, and more dramatically in the eyes of homozygous mice (Figure 8). This finding corroborated the partial or complete loss of photoreceptor layer segments observed by histological analysis (Figure 7). In retinal angiography, the degree of visibility of choroidal vessel structures decreased with increasing amounts of melanin in the RPE choroid interface [25] or, more importantly, with the presence of RPE cells in general.

Both FLA and ICGA revealed enhanced visibility of choroid structures in heterozygous and homozygous transgenic animals, in contrast to wild-type retinas (Figure 8). In particular, this indicates an at least partial degeneration of the RPE in transgenic mice, which results in the visibility of the choroidal vessels. Again, RPE malstructuring and partial loss was seen by histological inspection (Figure 7).

Finally, the lost integrity and regularity of retinal blood vessels and capillaries are also most probably caused by the retinal degeneration. With the advancing loss of retinal layers, blood vessels and capillaries lose their stabilizing environment and function, leading to malformed vessels with disturbed blood flow. The degeneration we observe in the adult retinas of hetero- and homozygous animals could occur for several reasons. As one possibility, the degeneration in later stages (six-month-old retinas) could simply be a secondary effect of already-reduced signaling in the P20 retinal neurons that display the rosette formations. The reduction or complete absence of neuronal function could lead to cell death. Another reason for degeneration could be the induction of cell death. Moreover, the arrangement of photoreceptors in rosettes seen in many animals not only leads to a hindered supply of glucose and oxygen, as well as an impaired homeostasis of the extracellular space, but also makes it difficult to establish functioning neuronal circuits. Previous reports have shown that SRF is not only required for proliferation, but that it also regulates the expression of antiapoptotic genes [40-42]. Furthermore, SRF has also been shown to play a crucial role in the survival of postnatal cortical neurons [43].

The SRF target gene *c-fos* has been reported to play a role in light-induced apoptotic cell death. Mice lacking functional c-Fos displayed less rod-photoreceptor loss as compared to wild-type littermates [44]. Further examination of retinal function in *c-fos*^−/−^ mice using ERG showed a reduction in retinal sensitivity due to a 22% reduction in number of rods and an overall lower rhodopsin content (25% reduced). The authors argue that apoptosis is induced normally by the bleaching of rhodopsin, but that the apoptotic cascade is interrupted downstream of this event in *c-fos*^−/−^ retinas due to the absence of Fos protein [45]. In this study, we have not addressed the role of *c-fos*; however, it is possible that the abnormal expression of *SRF-VP16* elicits an effect on *c-fos* expression, thereby affecting the resistance to light-induced damage and causing a more rapid degeneration. In fact, we do observe this in the retinas of *SRF-VP16* heterozygous and homozygous adult (P180) mice.

We conclude that there is an apparent correlation between the increases in transcript levels of both *SRF-VP16* and endogenous *Srf* and retinal rosette formation and degenerative processes in adult retinas. SRF represents a nuclear relay for signaling exerted through actin cytoskeletal dynamics [17]. In this function, SRF greatly impacts actin microfilament function and cellular behaviors directed by actin dynamics. Accordingly, dysregulation of SRF often causes phenotypes associated with impaired actin function [18]. Of interest regarding eye diseases, it has been found that corneal abnormalities caused by mutation of the *Destrin* gene, which encodes the actin depolymerizing factor (ADF), could be rescued by SRF depletion [46,47]. This study identified an actin-based, hierarchical functional relationship of *Destrin* and *Srf* in the corneal epithelium. In summary, we showed that *Gt(ROSA)26Sor^tm1(SRF-VP16)Antu^* transgenic mice display ectopic expression of SRF-VP16 and elevated levels of endogenous *Srf* in the retina. This overexpression of SRF activity correlates with structural distortions in the retinal layers, leading to the degeneration of photoreceptors and other neurons, as well as the degeneration of the RPE. In consequence, a severe loss of retinal function could be demonstrated by ERG. The severity of these effects is stronger in homozygous than in heterozygous animals, indicating a gene dosage dependence of the phenotypic effects caused by ectopic SRF activity. The reasons for and mechanisms of our findings are not fully understood so far. However, the findings suggest that SRF plays a role in retinal development and possibly also in the maintenance of retinal function in the adult animal. The next step in further investigation of the role of *Srf* in the mouse retina will be to cross the *Gt(ROSA)26Sor^tm1(SRF-VP16)Antu^* mouse strain with a Cre-expressing mouse line where Cre recombinase is driven by a retina-specific promoter. Null mutagenesis of *Srf* in the retina might provide further insight into the mechanisms by which SRF contribute to retinal function.
